# Stroke Deaths Profile and Its Subtypes in Brazil: Analysis Using Machine Learning

**DOI:** 10.5334/gh.1476

**Published:** 2025-10-03

**Authors:** Alessandro Rocha Milan de Souza, Letícia Martins Raposo, Glenda Corrêa Borges de Lacerda, Paulo Henrique Godoy

**Affiliations:** 1 Graduate Program in Neurology of the Center for Biological and Health Sciences, Federal University of the State of Rio de Janeiro (UNIRIO), Rio de Janeiro, Brazil; 2 Department of Quantitative Methods, Federal University of the State of Rio de Janeiro (UNIRIO), Rio de Janeiro, Brazil

**Keywords:** ischemic stroke, hemorrhagic stroke, mortality, machine learning

## Abstract

**Background:**

Brazil has one of the highest stroke rates in Latin America. It is important to understand the impact of other causes of death and sociodemographic factors, as this may contribute to a better comprehension of the stroke mortality process. Machine learning provides a means to explain this process.

**Objective:**

To investigate the stroke deaths profile and its subtype in Brazil using machine learning.

**Methods:**

This is a time series analysis where deaths mentioning stroke and other conditions were identified using individual death records from the country’s mortality information system (SIM) between 2000 and 2019. Strokes were grouped into the following subtypes: ischemic stroke (IS), hemorrhagic stroke (HS), and unspecified stroke (US). A decision tree model was built to identify the strongest factors distinguishing IS from HS.

**Results:**

There were 2,459,742 deaths mentioning stroke. There was a progressive increase in the number of deaths mentioning stroke over the study period. The most common type of stroke was US, accounting for more than 62% of deaths. Among HS deaths, hypertensive diseases were the most frequent group of associated causes (40.6%), while the most frequent group in subtypes IS and US was diseases of the respiratory system (48.30% and 42.30%, respectively). The decision tree analysis revealed that IS was more likely to occur in patients aged 60 years and over and in cases where respiratory diseases, endocrine diseases, arrhythmias, ischemic heart disease and heart failure were present. However, HS was more frequent in younger patients without these conditions but with nervous system diseases.

**Conclusions:**

The decision tree analysis identified the strongest factors distinguishing IS from HS, highlighting variables involved in each subtype of stroke-related death that can be recognized in clinical practice. These variables may also support the redistribution of deaths initially classified as unspecified stroke.

## Introduction

Stroke prevalence rates in Brazil vary between studies, regions and epidemiological contexts, ranging from 1.3% to 6.8% (1,2,3,4).

Brazil has the highest stroke mortality rate in Latin America, with rates being higher among women. Although there has been a decline in stroke mortality in the country in recent decades, rates remain high (5). A study by Souza et al. (in press) investigating trends in stroke mortality during the period 2000–2019 using data from the country’s mortality information system (SIM) reported an increase in cases of IS from 2015, which contrasts with global trends (6). However, the authors conclude that this increase may be related to improvements in reporting this cause of death in the SIM.

Strokes can be classified into two main groups: ischemic stroke (IS) and hemorrhagic stroke (HS). The latter is divided into intracerebral hemorrhage (ICH) and subarachnoid hemorrhage (SAH). Ischemic stroke is the most common type of stroke, accounting for around 65% of all cases worldwide, followed by ICH (26%) and SAH (9%) (7). ICH mortality can be up to two times higher than IS mortality (8). Risk factors differ according to stroke subtype, with systemic arterial hypertension and obesity being the leading factors for ICH and SAH, and systemic arterial hypertension and diabetes for IS (9).

Traditionally, the analysis of mortality has focused on the underlying cause of death. However, multiple causes of death, which include the underlying cause and contributing causes, provide a more complete picture of the process leading to death (10).

The analysis of multiple causes helps capture the true magnitude of contributing causes that may otherwise be hidden when only the underlying cause is considered, providing valuable information for the development of prevention strategies (11).

Machine learning algorithms have been widely employed in various fields of stroke medicine. One of the key benefits of machine learning is its ability to identify patterns and correlations in complex and big data sets, which can provide valuable insights into disease diagnosis, prognosis, and treatment (12). In addition, by automating the data analysis process, machine learning can reduce human bias (13). However, the use of these methods for analyzing multiple causes and associations with stroke subtypes is original in Brazil.

Considering the complexity of interactions between associated conditions and the sociodemographic factors that influence stroke, this study investigated the profile of deaths mentioning stroke in Brazil and the relationship between multiple causes of death and stroke subtype, using the machine learning decision tree.

## Materials and Methods

### Data source

National mortality data for the period 2000–2019 were obtained from the SIM using R’s microdatasus package (14).

### Identification of causes of death

Deaths mentioning stroke were identified using individual death records from the SIM, along with the other conditions and factors mentioned on the death certificate. Strokes were grouped into the following subtypes according to the International Statistical Classification of Diseases and Related Health Problems 10th Revision (ICD-10) codes (Supplement 1).

Cases where more than one stroke subtype was mentioned (approximately 2% of cases) were excluded to avoid ambiguity. Death records of individuals aged under 19 and where the year of death was missing were also excluded.

### Analysis of multiple causes

Multiple causes were defined as all causes mentioned on the death certificate, without distinguishing between underlying and contributing causes, as proposed by Santo (10).

The ICD-10 codes for the other causes of death related to the stroke subtypes were grouped according to the chapter. To facilitate data analysis and interpretation, the most frequent chapter (Chapter IX – Diseases of the circulatory system) was divided into groups that represent different types of circulatory diseases (Supplement 1).

Groups with a frequency of less than 1% were excluded. The chapter ‘Symptoms, signs and abnormal clinical and laboratory findings, not elsewhere classified’ (R00-R99) was also excluded because it does not include diseases, and the codes in this group may be regarded as garbage codes.

### Final dataset

The final dataset included the following variables: sex (female, male); race/skin color (yellow, white, indigenous, brown, black); marital status (single, consensual union, married, legally separated, widowed); education level (no education, 1–3 years, 4–7 years, 8–11 years, 12 years or more); place of death (home, hospital, other type of health facility, public thoroughfare, other); age group (19–39, 40–59, 60–79, 80 and over); region (North, Northeast, Midwest, Southeast, South); stroke subtype (IS, HS, US) and other causes of death mentioned on the death certificate.

### Statistical analysis

Descriptive statistics were used to describe the distribution of stroke subtypes (IS, HS and US) by demographic category, region, and period. The national and regional prevalence of each stroke subtype was also estimated.

### Decision tree

To identify the strongest factors distinguishing IS from HS, we built a decision tree model from the sample of death records mentioning IS and HS. Decision trees provide a clear visual representation of decision-making processes and are an ideal method for healthcare professionals because they facilitate the interpretation and practical application of clinical data. They are particularly useful for capturing complex nonlinear relationships between variables, providing an effective tool for making informed medical decisions. The analysis was performed using the CART (Classification and Regression Tree) algorithm (15), implemented using the rpart package in R (16).

Decision trees sequentially apply a set of rules that split a predictor variable into a binary response. The splitting criterion is based on the concept of ‘purity’, where a node is pure if all its elements belong to a single class. When a node is impure, the algorithm splits the node to minimize impurity.

For model assessment and validation purposes, the dataset was divided into training and test sets using a 70/30 split. The complexity parameter (cp) was set at values ranging from 0.001 to 0.1 using the caret package train function (17) in R and 5-fold cross-validation and area under the Receiver Operating Characteristic curve (AUC-ROC) as an evaluation metric. The ideal ‘cp’ value for the decision tree model was 0.001. This parameter is essential to prune decision tree complexity and avoid overfitting, thus controlling cost-complexity.

The graphical representation of the decision tree was built using the rpart.plot package (18) in R. Model performance was assessed using the test set based on the AUC-ROC and interpreted following the guidelines proposed by Hosmer and Lemeshow (19): ROC = 0.5 is considered no better than chance, 0.6–0.69 indicates poor discrimination, 0.7–0.79 indicates acceptable (reasonable) discrimination, 0.8–0.89 indicates good (excellent) discrimination, and 0.9–1.0 indicates outstanding discrimination.

## Results

There were 2,459,742 deaths mentioning stroke between 2000 and 2019. The period that accounted for the largest proportion of deaths mentioning stroke was 2015–2019. The regions with the highest and lowest number of deaths mentioning stroke were the Southeast and North, respectively. The following sociodemographic groups accounted for the highest proportion of overall deaths in their respective categories: the 60–79 age group, white people, married people, and people with less than three years of education. Men accounted for 50.7% of overall deaths. The most common place of death was a hospital, followed by at home (Table 1). The most common type of missing data was education.

**Table 1:** Sociodemographic characteristics of deaths mentioning stroke (overall and by subtype).


	OVERALL	STROKE SUBTYPE		

PERIOD AND SOCIODEMOGRAPHIC CHARACTERISTIC	N = 2,459,742^1^	HEMORRHAGIC N = 472,052^1^	ISCHEMIC N = 470,401^1^	UNSPECIFIED N = 1,517,289^1^

**Period**				

2000–2004	567,101 (23.1%)	103,235 (21.9%)	93,021 (19.8%)	370,845 (24.4%)

2005–2009	605,534 (24.6%)	111,169 (23.6%)	103,603 (22.0%)	390,762 (25.8%)

2010–2014	635,649 (25.8%)	122,400 (25.9%)	121,474 (25.8%)	391,775 (25.8%)

2015–2019	651,458 (26.5%)	135,248 (28.7%)	152,303 (32.4%)	363,907 (24.0%)

**Region**				

Midwest	145,622 (5.9%)	32,010 (6.8%)	31,079 (6.6%)	82,533 (5.4%)

Northeast	643,693 (26.2%)	107,402 (22.8%)	88,496 (18.8%)	447,795 (29.5%)

North	126,764 (5.2%)	24,912 (5.3%)	16,748 (3.6%)	85,104 (5.6%)

Southeast	1,110,449(45.1%)	236,795 (50.2%)	234,149 (49.8%)	639,505 (42.1%)

South	433,214 (17.6%)	70,933 (15.0%)	99,929 (21.2%)	262,352 (17.3%)

**Age group (years)**				

19–39	76,438 (3.1%)	46,227 (9.8%)	9,306 (2.0%)	20,905 (1.4%)

40–59	409,258 (16.6%)	161,417 (34.2%)	62,395 (13.3%)	185,446 (12.2%)

60–79	1,145,977 (46.6%)	187,589 (39.7%)	226,729 (48.2%)	731,659 (48.2%)

≥ 80	828,069 (33.7%)	76,819 (16.3%)	171,971 (36.6%)	579,279 (38.2%)

**Sex**				

Female	1,212,314 (49.3%)	233,129 (49.4%)	238,844 (50.8%)	740,341 (48.8%)

Male	1,247,168 (50.7%)	238,877 (50.6%)	231,517 (49.2%)	776,774 (51.2%)

DC^2^	260 (0.01%)	46	40	174

**Race/skin color**				

Yellow	16,147 (0.7%)	3,742 (0.9%)	3,261 (0.7%)	9,144 (0.7%)

White	1,301,687 (57.0%)	241,956 (55,3%)	280,430 (63,5%)	779,301 (55,6%)

Indigenous	3,742 (0.2%)	690 (0.2%)	531 (0.1%)	2,521 (0.2%)

Brown	749,505 (32.8%)	151,030 (34.5%)	120,789 (27.3%)	477,686 (34.1%)

Black	210,581 (9.2%)	39,899 (9.1%)	36,803 (8.3%)	133,879 (9.5%)

DC^2^	178,080 (7.24%)	34,735	28,587	114,758

**Marital Status**				

Single	453,161 (19.9%)	111,850 (25.7%)	76,770 (17.5%)	264,541 (18.8%)

C Union^3^	34,925 (1.5%)	8,808 (2.0%)	5,607 (1.3%)	20,510 (1.5%)

Married	953,753 (41.9%)	196439 (45.2%)	180,205 (41.0%)	577,109 (41.1%)

Separated	113,147 (5.0%)	29,609 (6.8%)	23,975 (5.5%)	59,563 (4.2%)

Widowed	723,502 (31.8%)	88,254 (20.3%)	153,305 (34.9%)	481,943 (34.3%)

DC^2^	181,254(7,4%)	37,092	30,539	113,623

**Education level**				

None	503.908 (28.7%)	52,763 (16.2%)	80,089 (23.8%)	371,056 (33.9%)

1–3 y	587,334 (33.4%)	98,464 (30.2%)	117,877 (35.1%)	370,993 (33.9%)

4–7 y	394,355 (22.5%)	89,701 (27.5%)	80,705 (24.0%)	223,949 (20.5%)

8–11 y	188,750 (10.7%)	58,261 (17.9%)	39,862 (11.9%)	90,627 (8.3%)

≥12 y	81,621 (4.6%)	26,748 (8.2%)	17,339 (5.2%)	37,534 (3.4%)

DC^2^	703,774(28.61%)	146,115	134,529	423,130

**Place of death**				

At home	419,374 (17.1%)	26,532 (5.6%)	53,001 (11.3%)	339,841 (22.4%)

Hospital	1,917,378 (78.0%)	422,563 (89.6%)	399,274 (84.9%)	1,095,541 (72.3%)

Other^4^	73,706 (3.0%)	9,861 (2.1%)	11,493 (2.4%)	52,352 (3.5%)

Pub thoroughfare^5^	14,438 (0.6%)	7,191 (1.5%)	901 (0.2%)	6,346 (0.4%)

Other	31,781 (1.3%)	5,427 (1.2%)	5,400 (1.1%)	20,954 (1.4%)

DC^3^	3,065(0.13%)	478	332	2,255


^1^Total number. ^2^ Unknown. ^3^Consensual union. ^4^Other type of health facility. ^5^Public thoroughfare.

The most common type of stroke was US, accounting for 62% of all deaths mentioning stroke. The number of deaths mentioning IS and HS increased over the study period, while the number mentioning US remained relatively stable. HS accounted for a higher number of deaths than IS in all regions except the South. In the Northeast and North, the US accounted for a larger proportion of deaths than IS and HS. Younger individuals (≤59 years) represented 44% of HS deaths, whereas the majority of IS and US deaths occurred in older age groups (≥60 years). Women accounted for a higher proportion of deaths mentioning IS than men.

Stroke subtypes showed distinct sociodemographic patterns. White individuals and married people predominated across all stroke subtypes, while widows/widowers were more frequent in IS and US, and single status in HS. Low education was strongly associated with IS mortality. Hospitals were the main place of death overall, while home deaths predominated in the US subtype (Table 1).

**Figure 1:**
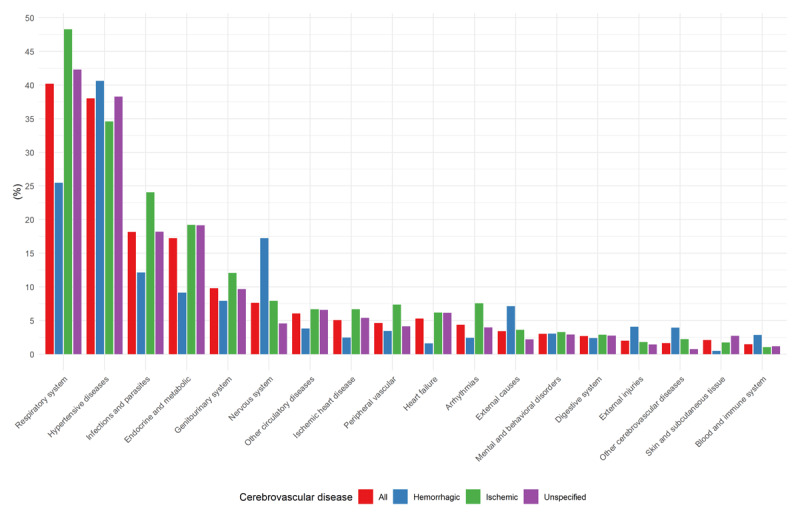
Frequency of groups of causes of death in deaths mentioning stroke (overall and by subtype).

Overall, the most frequent causes of death associated with stroke were respiratory diseases (40.2%), followed by hypertensive ones (38%). In HS, hypertensive diseases predominated, whereas in IS and US, respiratory diseases were most common. Among IS deaths, endocrine diseases (19.2%), infections (24.1%), arrhythmia (7.6%), ischemic heart disease (6.7%), and heart failure (6.2%) were notable, compared to lower frequencies in HS. Conversely, HS showed higher proportions of hypertensive (40.6%) and nervous system diseases (17.2%). The patterns for US largely mirrored IS, except for arrhythmias, peripheral vascular diseases, and hypertensive diseases, where US resembled HS (Figure 1).

**Figure 2:**
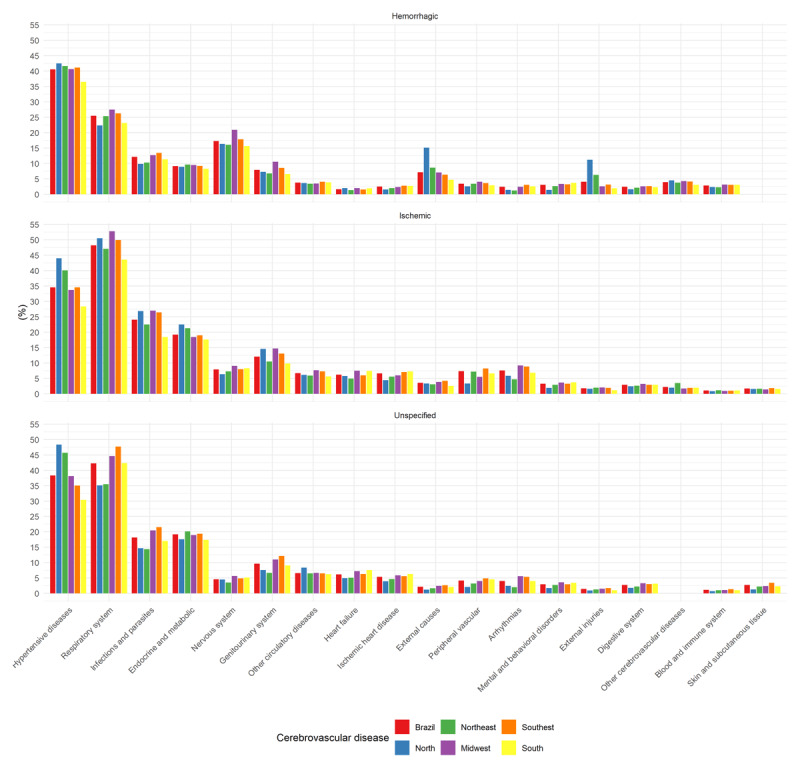
Frequency of groups of causes of death among deaths mentioning stroke by subtype and region.

Across all regions, hypertensive diseases were the most frequent causes of death in HS and IS, followed by respiratory diseases. In the North, hypertensive diseases predominated across all subtypes. In the Midwest, respiratory and nervous system diseases were more frequent in HS and IS. In HS, deaths from external causes (15.1%) and from injury, poisoning, and related conditions (11.2%) were also highest in the North (Figure 2).

## Decision Tree Analysis

**Figure 3:**
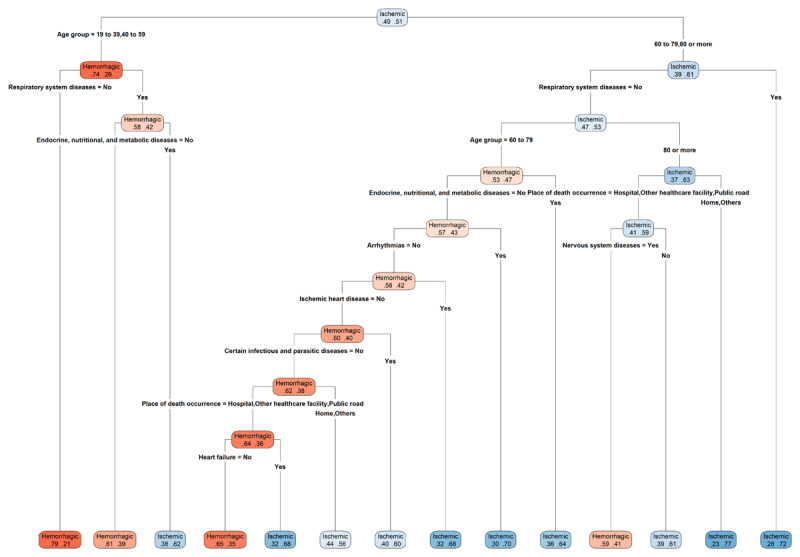
Decision tree for hemorrhagic and ischemic stroke. The percentages on the left represent hemorrhagic stroke and the colors represent magnitude.

The decision tree model (Figure 3) classifying deaths mentioning HS and IS was built from a dataset containing 442,451 observations. The model’s AUC value was 0.727 (95% CI 0.724–0.729), indicating reasonable performance. At the root node, the distribution of deaths was balanced (HS: 49%, IS: 51%). The first partition was determined by age, with 74% of individuals aged 19–59 years being classified as HS. Within this subgroup, the proportion of deaths mentioning HS was considerably higher in the absence of diseases of the respiratory system than in cases where this group was present. Subsequently, cases were further divided according to the presence of endocrine, nutritional, and metabolic diseases. In this branch, the proportion of deaths mentioning HS was markedly higher among cases without these comorbidities compared to those in which they were present (61% versus 38%) (Figure 3).

Among individuals aged 60 years and older, most cases were classified as IS, particularly when respiratory diseases were present. In the absence of respiratory diseases, classification varied according to age, with older individuals more frequently associated with IS. When the analysis was further stratified by place of death—occurring in hospitals, other healthcare facilities, or public thoroughfares—without mention of nervous system diseases, cases tended to be classified as IS, whereas the presence of such diseases was more often associated with HS. Deaths occurring at home or in other non-institutional settings were predominantly classified as IS (Figure 3).

In the 60–79 age group without respiratory diseases, deaths were predominantly classified as HS. Within this subgroup, the presence of endocrine, nutritional, and metabolic diseases was associated with a higher frequency of IS. In the absence of these conditions, IS predominated when arrhythmia or ischemic heart disease was present. Conversely, when these cardiovascular conditions were absent, but certain infectious or parasitic diseases were reported, the frequency of IS declined. In a hospital, health facility, or public settings, the presence of heart failure was associated with a predominance of IS, whereas in its absence HS was more frequent (Figure 3).

## Discussion

The period with the largest proportion of deaths mentioning stroke was 2015–2019. A previous study investigating trends in stroke mortality based on underlying cause (in press) reported a spike during this period (6). The authors explained that this increase resulted from a reduction in US over the period, due to improvements in SIM data quality.

The Southeast continues to account for the highest number of deaths in Brazil. Higher mortality rates in more developed states may be explained by the fact that chronic conditions have a greater influence on the mortality profile in these more populous regions (20).

US remains the most common type of stroke despite a decline in the number of ill-defined causes of death across the country (21). In the Northeast and North, US accounted for a higher proportion of deaths mentioning stroke (29.5% and 5.6%, respectively) than IS and HS. Garritano et al.’s findings corroborated the same, representing more than 15% of deaths in older adults in the North (22), while Jorge et al. reported that US accounted for over 20% of stroke deaths in the North and Northeast (23). Both studies were based on data from the SIM, although different ICD-10 codes were applied, which may explain the variation in reported proportions. Key problems in these regions include poor access to health care, owing to their huge geographic area and cultural factors influencing health behavior (24).

Deaths from US remain a challenge for estimating stroke mortality, requiring redistribution efforts. Direct methods (25), such as reviewing archived death certificates or registry books, though accurate, are labor-intensive, costly, and operationally complex (26). For this reason, indirect approaches—including those adopted by the GBD study—have been employed (27). Other alternatives, such as redistributing according to ICD-10-chapter proportions, have been suggested but remain unsatisfactory (28).

Our findings show that younger adults account for a larger proportion of deaths mentioning HS, while older adults account for a larger proportion of cases mentioning IS and US. These findings corroborate the results of a study by De Moraes et al., who found that 76% of deaths from HS in the South and Southeast of Brazil were among younger patients (10–49 years) (29). Taken together, the two studies converge in showing the predominance of HS in younger adults. However, the differences in age range and case definition—particularly the exclusion of US in De Moraes et al.—prevent a direct comparison of results.

Our results show that the number of white people afflicted by stroke was more than that of black and brown people combined across all stroke subtypes. This can be explained by the fact that the population in the regions that account for the highest numbers of deaths from stroke (the Southeast and South) is predominantly white (30). However, international literature reports that stroke incidence (31), the prevalence of risk factors such as hypertension and diabetes, and levels of cardiovascular disease biomarkers such as lipoprotein A (32) tend to be higher among black people. In addition, this group also experiences inequalities across various social determinants of health, tending to have lower socioeconomic status and education level and poorer access to health care than white people (33). In a study of race-adjusted stroke deaths in Brazil in 2010 based on underlying cause, Lotufo and Bensenor (34) found that mortality was higher among black people. The difference between our results and the findings of the above study may be explained by the fact that the current study investigated stroke deaths based on multiple causes rather than just underlying causes. Indigenous people accounted for the lowest proportion of deaths in our sample, which is consistent with the findings of the previous study (35), though this may also reflect underreporting and barriers to health care access rather than a true lower disease burden.

The fact that the proportion of deaths is lower in individuals with a higher level of education may be explained by higher income and better access to goods and services among this group, including health care (36). However, it is important to recognize potential confounding by socioeconomic context, as education may also correlate with regional differences in diagnosis and reporting practices.

The fact that a higher proportion of deaths mentioning US occurred at home may be explained by diagnostic challenges and the absence of a death verification service (37). Challenges in defining death in non-healthcare settings were highlighted by Santo in a study investigating ill-defined deaths in Brazil in 2003, showing that more than half of such deaths were unattended (38). However, the fact that the proportion of deaths mentioning US in our study were in-hospital deaths may be partially explained by poor diagnosis skills, given that there was no significant change in this percentage with the presence of a CT scan (39).

Education was the sociodemographic data with the highest degree of incompleteness, consistent with findings from the states of Rio Grande do Sul (40), Fortaleza (41), Bahia (42), and Pernambuco (43). As an indicator of social vulnerability (44), education is particularly relevant, although other variables, such as race, may also serve as proxies.

Studies show that up to 30% of death certificates contain significant errors that could be avoided with proper training (45). According to Mendonça et al. (46), the main causes of incomplete data include insufficient dissemination of instructions, lack of information for proper registration, limited physician knowledge regarding detailed cause-of-death reporting, and low awareness of the importance of complete and accurate completion of the forms for SIM data quality. These limitations highlight a potential source of bias in our findings, as differential patterns of underreporting by a region or sociodemographic group may have influenced the observed associations.

The most frequent group of causes of death in deaths mentioning stroke at national level and across regions was diseases of the respiratory system, followed by hypertensive diseases, ill-defined causes, infectious diseases and endocrine diseases. A study by Santo and Pinheiro (47) in São Paulo showed that the primary causes related to stroke deaths were respiratory diseases (41.8%), hypertension (37.8%), and ill-defined causes (26.3%). A study in the state of Paraná by Furukawa et al. (48) reported that the leading causes of death related to stroke deaths were diseases of the circulatory system (52.2%), diseases of the respiratory system (31.1%), and ill-defined causes (27%), while an investigation in Belo Horizonte by Ishitani and França (49) found that the main causes were hypertensive diseases (33.8%) and respiratory system diseases (28.1%). While these results show general agreement across different contexts, they also reveal variability in the ranking of comorbid conditions, which may reflect both true regional differences in health profiles and disparities in diagnostic and reporting practices.

The high proportion of respiratory diseases recorded as associated causes could reflect complications of stroke, such as aspiration pneumonia, with prevalence of post-stroke dysphagia standing at around 40% (50). Stroke patients with dysphagia have an 8.5-fold higher risk of death, a higher risk of pneumonia and higher in-hospital costs (51). The present study found that respiratory system diseases were more frequent in deaths mentioning IS. In contrast, a meta-analysis by Banda et al. (50) investigating the prevalence of dysphagia and risk of pneumonia and mortality in acute stroke patients found that prevalence of dysphagia was higher in patients with HS (OR 1.52 [95%CI, 1.13–2.07]). Our findings show that pneumonia and respiratory failure account for 90% of the mentions in the group diseases of the respiratory system (data not shown), underscoring their central role as complications of stroke. While this finding highlights the clinical relevance of respiratory complications, caution is needed when interpreting death certificate data. The absence of clinical validation means that some mentions of pneumonia or respiratory failure may represent presumptive diagnoses rather than confirmed complications, leading to possible over-attribution.

Laurenti (52) and Santo (10) reported that primary arterial hypertension was related to stroke in 75.9% and 57.4% of deaths respectively. In the present study, primary arterial hypertension was more frequent in deaths mentioning HS than in those mentioning IS (40.6% versus 34.6%). The INTERSTROKE study (53) also found that the association between hypertension and stroke was stronger for HS than for IS.

While certain diseases were mentioned together with stroke on the death certificate, causal relationships cannot be inferred. Some of these diseases may be risk factors, while others may be complications related to the underlying cause (54). Our findings suggest that this may be the case with primary hypertension and diseases of the respiratory system, which were the two most prevalent causes at national level and across regions, underscoring their clinical and epidemiological significance. Although the inability to disentangle these relationships in mortality data represents a limitation, our use of a decision tree helped to highlight the most influential predictors in a transparent way.

Endocrine diseases, arrhythmias, ischemic heart disease and heart failure were more frequent in deaths mentioning IS than among those mentioning HS. In a study examining the relationship between vascular risk factors and stroke types in native Black Africans, Owolabi and Agunloye (55) found that the association between arrhythmias, heart failure, diabetes, and stroke was stronger for IS than for HS. However, caution is warranted, as differences across studies may reflect not only biological mechanisms but also disparities in diagnostic capacity, coding practices, and the availability of detailed clinical information.

Importantly, the frequency of hypertensive diseases was highest in the North and Northeast, across all stroke subtypes. Although the 2019 National Health Program (PNS) (56) shows that the North and Northeast are not the regions with the highest proportion of people diagnosed with hypertension, access to medications dispensed by the popular pharmacy was lowest in these regions (34.8% and 38% respectively, compared to 45% nationally). Furthermore, the percentage of hypertensive patients undergoing drug therapy for the condition is lower in these regions (57). This gap between prevalence and treatment access reflects broader structural inequalities in health care provision. Studies of the prevalence of chronic diseases such as primary arterial hypertension show that rates are higher among economically disadvantaged groups (58). Furthermore, in an ecological study investigating the relationship between socioeconomic indicators and cardiovascular disease mortality in 98 municipalities in Brazil, Ishitani et al. (36) found an inverse correlation between death from cardiovascular, cerebrovascular, and hypertensive diseases and income, and a direct association with poverty and precarious living conditions. These findings reinforce that stroke mortality disparities are rooted in social determinants of health.

In the North, external causes of morbidity, mortality, injury, poisoning, and certain other consequences were more frequent in deaths mentioning HS than in deaths mentioning IS. According to the 2019 National Health Survey (NHS), the proportion of individuals who drove a motor vehicle after drinking was 17% nationally, ranging from 14.8% in the South and Southeast to 23.4% in the North. This rate was higher in rural areas (22.5% compared to 16.2% in urban areas) (56). The higher frequency of group injury, poisoning, and certain other consequences of external causes in the North may therefore be explained by higher rates of head trauma due to accidents in this region. At the same time, regional disparities in road safety infrastructure, enforcement of traffic laws, and access to trauma care may also contribute to these differences. Thus, the observed association between HS mortality and external causes in the North likely reflects a complex interplay of behavioral, structural, and health system factors.

The decision tree revealed complex patterns in the classification of deaths mentioning HS and IS, revealing that the distinction between the two depends on complex interactions between age and the presence of respiratory diseases and other comorbidities. Age group was the strongest initial factor, with younger individuals (19–59 years) being classified predominantly as HS and older individuals (60 years and older) predominantly as IS. These findings are consistent with those of Bernal et al., who investigated the incidence of hospitalization and mortality due to stroke in young adults in the Southeast and South of Brazil, finding that HS accounted for 76% of the 78,123 hospitalizations due to stroke in this group between 2008 and 2018 (29).

In older adults, the frequency of IS was higher in cases with the presence of respiratory system diseases, while in younger adults the frequency of HS was higher in cases not mentioning this group of causes of death. This may be partially explained by the increased risk of aspiration pneumonia in older IS patients with dysphagia. For example, Arnold et al. reported that 20.7% of IS patients at a Swiss tertiary center developed dysphagia, and 13.3% of those with dysphagia died within three months due to respiratory complications (51). These findings may partially explain the higher frequency of IS in cases mentioning respiratory system diseases among older adults in our study.

Other strong factors included endocrine, nutritional and metabolic diseases, arrhythmias, and place of death. Cases mentioning endocrine, nutritional and metabolic diseases were predominantly IS, regardless of age group. These findings are consistent with the findings of a collaborative meta-analysis of 102 prospective studies showing that adjusted hazard ratios with diabetes was 2.27 (1.95–2.65) for IS and 1.56 (1.19–2.05) for HS (59). In patients aged over 60, arrhythmias, ischemic heart disease, and heart failure were more frequent in deaths mentioning IS, echoing evidence from the Framingham study (60) that showed a 5-fold excess of stroke in the presence of atrial fibrillation, and from Adelborg et al. (61), who found an increase in the stroke rate ratio among patients with heart failure. Using autopsy data, Gongora-Rivera et al. (62) found that myocardial infarction was present in 40.8% of 251 patients who died from IS. These patterns emphasize the central role of cardiometabolic comorbidities in shaping the mortality profile of IS in older adults.

Place of death was also a strong factor, with deaths at home being predominantly IS, especially among older adults. It is therefore possible that most US deaths at home among individuals aged 80 and over are actually IS.

Unlike traditional regression-based approaches, which were employed by Santo and Pinheiro (47), the machine learning decision tree enabled the visualization of how multiple factors interact hierarchically to influence whether a death is more likely to be IS or HS. This interpretability is clinically and epidemiologically valuable: it highlights that stroke mortality is not determined by isolated variables, but by combinations of sociodemographic and clinical conditions. Such insights can guide health systems in tailoring prevention and intervention strategies—for instance, prioritizing dysphagia screening in elderly IS patients, intensifying hypertension control in regions with limited treatment access, and improving diagnostic services for deaths occurring outside hospitals.

Although the decision tree achieved acceptable discriminant validity, suggesting it was effective in identifying relevant associations, some caution is warranted. Mortality data are subject to underreporting, coding errors, and a lack of clinical validation (e.g., imaging or treatment data), which limit causal inference and may inflate or obscure certain associations. Nevertheless, by leveraging a nationwide dataset covering millions of records over two decades, this study provides representative evidence on stroke mortality in Brazil. The decision tree approach, by exploring interactions between variables rather than assessing them in isolation, adds a novel and practical dimension to the analysis, offering guidance for public health interventions and underscoring the need for future studies linking mortality and clinical data to refine risk prediction and prevention policies.

## Conclusions

The analysis of multiple causes allowed a comprehensive characterization of deaths mentioning ischemic and hemorrhagic stroke, identifying key contributing conditions and providing relevant evidence for care planning. The decision tree highlighted factors and comorbidities most strongly associated with IS and HS, offering an interpretable framework that can guide both clinical practice and public health strategies. In addition, these findings may support more accurate redistribution of deaths initially coded as unspecified stroke, thereby improving the quality of stroke mortality estimates in Brazil.

## Additional File

The additional file for this article can be found as follows:

10.5334/gh.1476.s1 Supplementary files. **Table 1:** ICD-10 codes according to stroke subtypes. **Table 2:** Groups of Circulatory System Diseases (Chapter IX). **Figure 1:** Flowchart of death identification in mortality databases.
