# Supplementary material for: Stroke Deaths Profile and Its Subtypes in Brazil: Analysis Using Machine Learning
Source: Glob Heart. 2025 Oct 3;20(1):85. doi: 10.5334/gh.1476 (PMC12493032; doi:10.5334/gh.1476)
Supplement: Supplementary files. — Table 1: ICD-10 codes according to stroke subtypes. Table 2: Groups of Circulatory System Diseases (Chapter IX). Figure 1: Flowchart of death identification in mortality databases. [file gh-20-1-1476-s1.pdf]

Supplementary Table 1. ICD-10 codes according to stroke subtypes and Chapter IX groups

Stroke subtype	ICD-10 codes
Ischemic stroke (IS)	I63, I67.3, I67.8, I69.3
Hemorrhagic stroke (HS)	I60, I61, I62, I69.0, I69.1, I69.2
Unspecified stroke (US)	I64, I67.9, I69.4, I69.8

Supplementary classification of other causes of death (ICD-10, Chapter IX – Diseases of the circulatory system)

Group	ICD-10 codes
Hypertensive diseases	I10–I15
Ischemic heart diseases	I20–I25
Heart failure	I50
Cardiac arrhythmias	I48–I49
Peripheral vascular diseases	I70–I99
Other circulatory system diseases not elsewhere classified*	I00–I09, I26–I46, I51–I59

*Excluding stroke-related codes (I60–I69), which were classified separately according to the three stroke subtypes above.

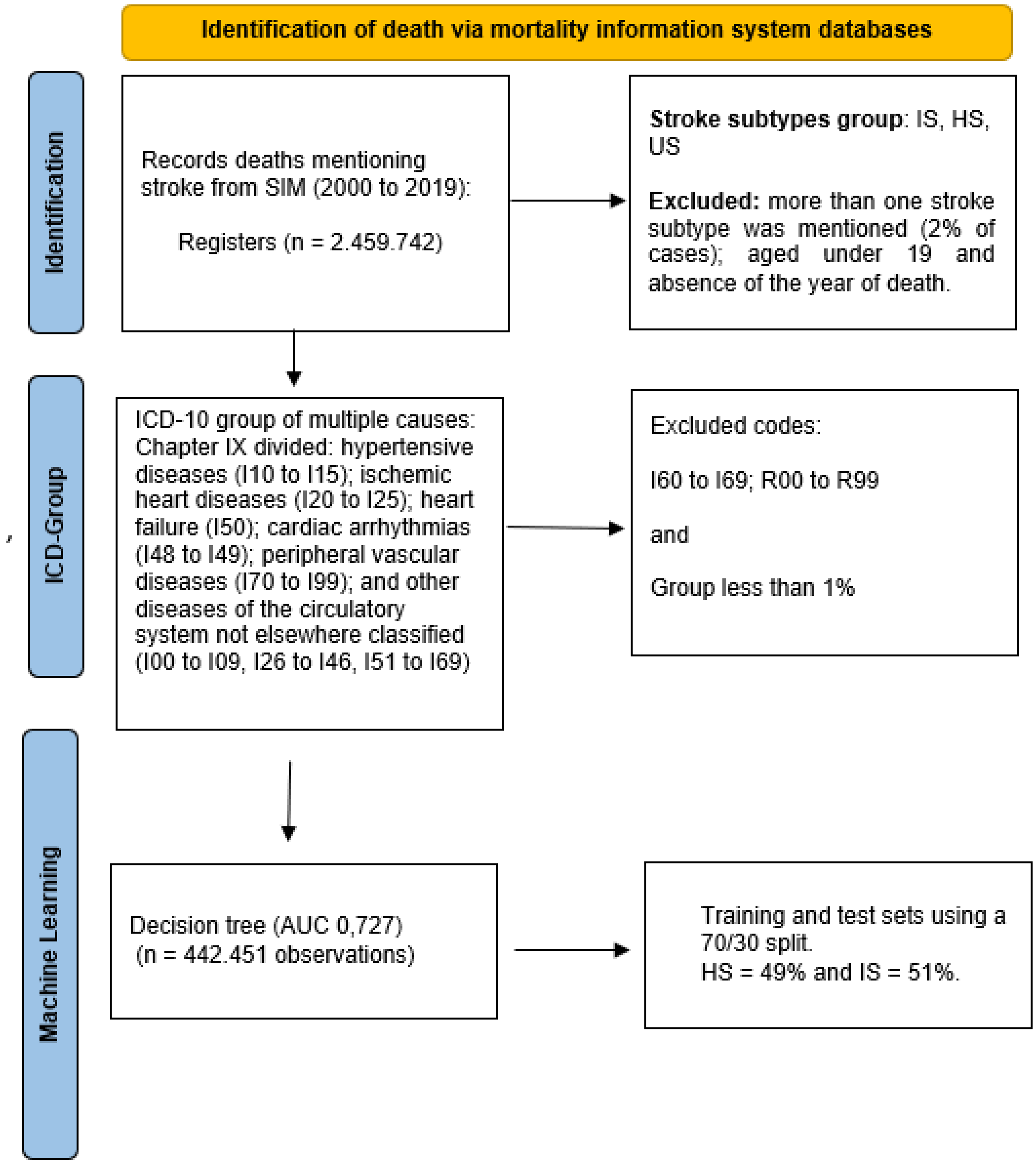

Source: Page MJ, et al. BMJ 2021;372:n71. doi: 10.1136/bmj.n71.

This work is licensed under CC BY 4.0. To view a copy of this license, visit https://creativecommons.org/licenses/by/4.0/
